# Arteria Lusoria: An Unusual Cause of Dysphagia

**DOI:** 10.7759/cureus.81092

**Published:** 2025-03-24

**Authors:** Brianna Castellano, Riya Kumar, Maria Buencamino, Kristel Sibaja, Alejandro Biglione

**Affiliations:** 1 Medicine, Dr. Kiran C. Patel College of Osteopathic Medicine, Nova Southeastern University, Fort Lauderdale, USA; 2 Internal Medicine, Wellington Regional Medical Center, Wellington, USA

**Keywords:** acute dysphagia, arteria lusoria, dysphagia, dysphagia in the elderly, rare cause of dysphagia

## Abstract

Arteria lusoria is an uncommon anatomical variant in which the right subclavian artery originates from the descending aorta rather than the brachiocephalic trunk. The vascular anomaly causes compression of the esophagus and can lead to dysphagia. Differential diagnosis includes other causes of dysphagia such as neurological, functional, or structural disorders. Diagnosis is achieved by radiological modalities, and treatment with dietary modifications is usually successful. However, severe or refractory cases require surgical intervention. This study presents the case of a 72-year-old woman with intermittent dysphagia who was found to have an aberrant origin of the right subclavian artery on CT angiography (CTA) of the chest. Arteria lusoria remains a diagnostic challenge. This paper describes the clinical manifestations, diagnostic approach, and management of arteria lusoria.

## Introduction

Arteria lusoria, also known as aberrant right subclavian artery (ARSA), is a rare anatomical variant characterized by the origin of the right subclavian artery left of midline, traversing a retroesophageal course [1]. This anatomical aberration, though infrequent, can lead to compression of adjacent structures within the thoracic cavity, resulting in various clinical presentations. When the esophagus is compressed, it can result in dysphagia that has been called dysphagia lusoria. We report a case of a 72-year-old woman with dysphagia unexpectedly found to be arteria lusoria, emphasizing the importance of recognizing this anatomical variant as a cause of dysphagia.

## Case presentation

The patient was a 72-year-old woman with a past medical history (PMH) of hypertension, chronic obstructive pulmonary disease (COPD), chronic kidney disease stage 3, type 2 diabetes, breast cancer, dyslipidemia, and depression. She presented to the emergency room (ER) complaining of shortness of breath associated with cough, wheezing, and scant sputum production for three days. She denied fever, chills, chest pain, weight loss, paroxysmal dyspnea, abdominal pain, nausea, vomiting, and heartburn, but admitted to intermittent dysphagia to solids that had been present for years. She denied alcohol use but she was a current smoker with a 45-pack-year history from the age of 20. Her surgical history is significant for a right breast lumpectomy. Her family history was remarkable for her deceased mother with a history of breast cancer. On physical examination her vitals showed temperature 98.4 degrees Fahrenheit, pulse 109 beats/minute, blood pressure (BP) of 143/84 mmHg, respiratory rate of (RR) of 18 breaths/minute, oxygen saturation 92% on room air, and BMI of 23.88. The patient was awake, alert, and orientated in person, time, and place, and in moderate respiratory distress. Neck was without jugular venous distension and there were no palpable neck masses. Chest was normal to inspection, without accessory muscle use, with bilateral generalized wheezing. The abdomen was soft and non-tender to palpation. No abdominal masses or organomegaly were noted. The bowel sounds were normal. There was no lower extremity edema noted. A complete blood count (CBC) and metabolic panel were taken on admission (Tables 1, 2). 

**Table 1:** Initial CBC CBC: Complete blood count

Lab	Initial Value	Normal Range
White Blood Cell Count	11.97x10^3^/mcL	4.50x10^3^/mcL - 10.50x10^3^/mcL
Red Blood Cell Count	4.43x10^6^/mcL	4.40x10^6^/mcL - 6.15x10^6^/mcL
Hemoglobin	14.2 gm/dL	14.0 gm/dL - 18.0gm/dL
Hematocrit	41.6%	40.0% - 54.0%
Mean Corpuscular Volume	93.9fL	81.0 fL - 96.0 fL
Mean Corpuscular Hemoglobin	32.1 pg	27.0 pg - 34.0 pg
Platelet Count	261x10^3^/mcL	150x10^3^/mcL - 450x10^3^/mcL

**Table 2:** Initial CMP BUN: Blood urea nitrogen; CMP: Complete metabolic panel

Lab	Initial Value	Normal Range
Glucose	333 mg/dL	74 mg/dL - 106 mg/dL
Sodium	134 mmol/L	135 mmol/L - 148 mmol/L
Potassium	3.5 mmol/L	3.6 mmol/L - 5.2 mmol/L
Chloride	95 mmol/L	95 mmol/L - 110 mmol/L
Bicarbonate	30 mEq/L	21 mEq/L - 32 mEq/L
BUN	35 mg/dL	7 mg/dL - 18 mg/dL
Creatinine	1.24 mg/dL	0.7 mg/dL - 1.3 mg/dL

**Figure 1:**
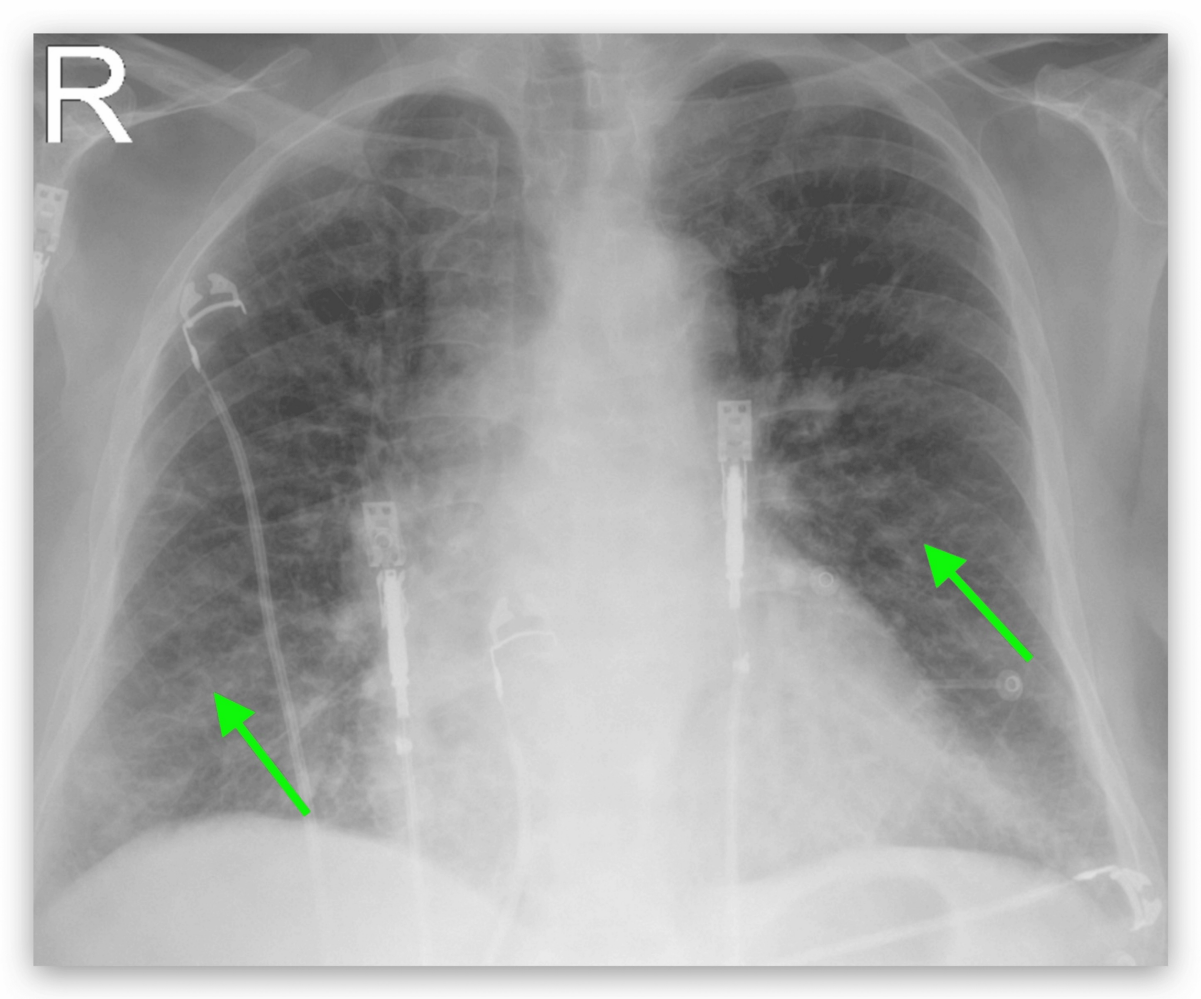
Chest X-ray showed diffuse interstitial infiltrates (green arrows)

**Figure 2:**
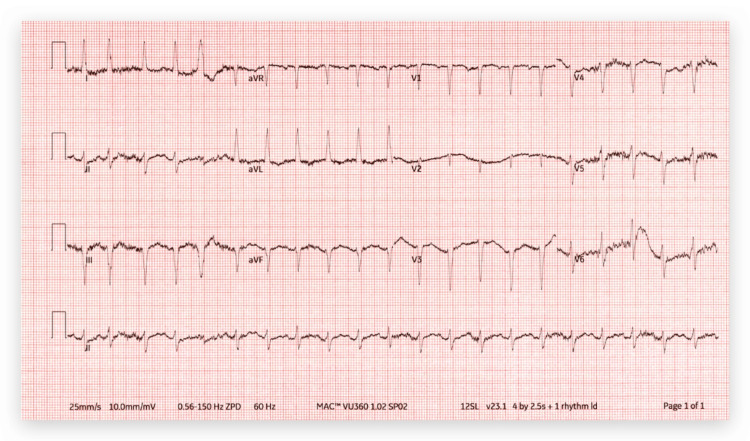
EKG showed sinus tachycardia with premature supraventricular complexes and left axis deviation EKG: Electrocardiogram

**Figure 3:**
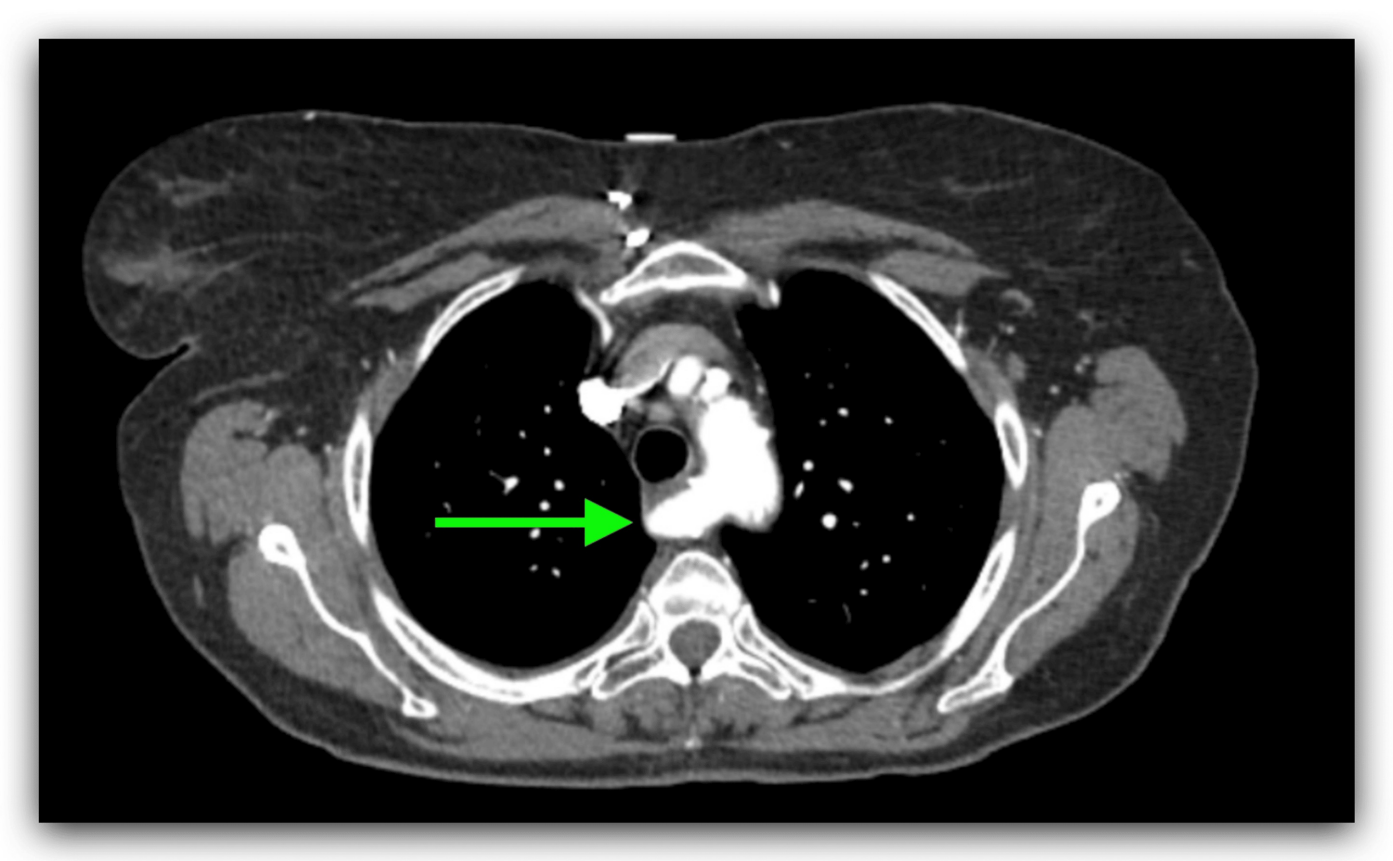
CTA of the chest with contrast showed aberrant origin of the right subclavian artery (green arrow) CTA: CT angiography

**Figure 4:**
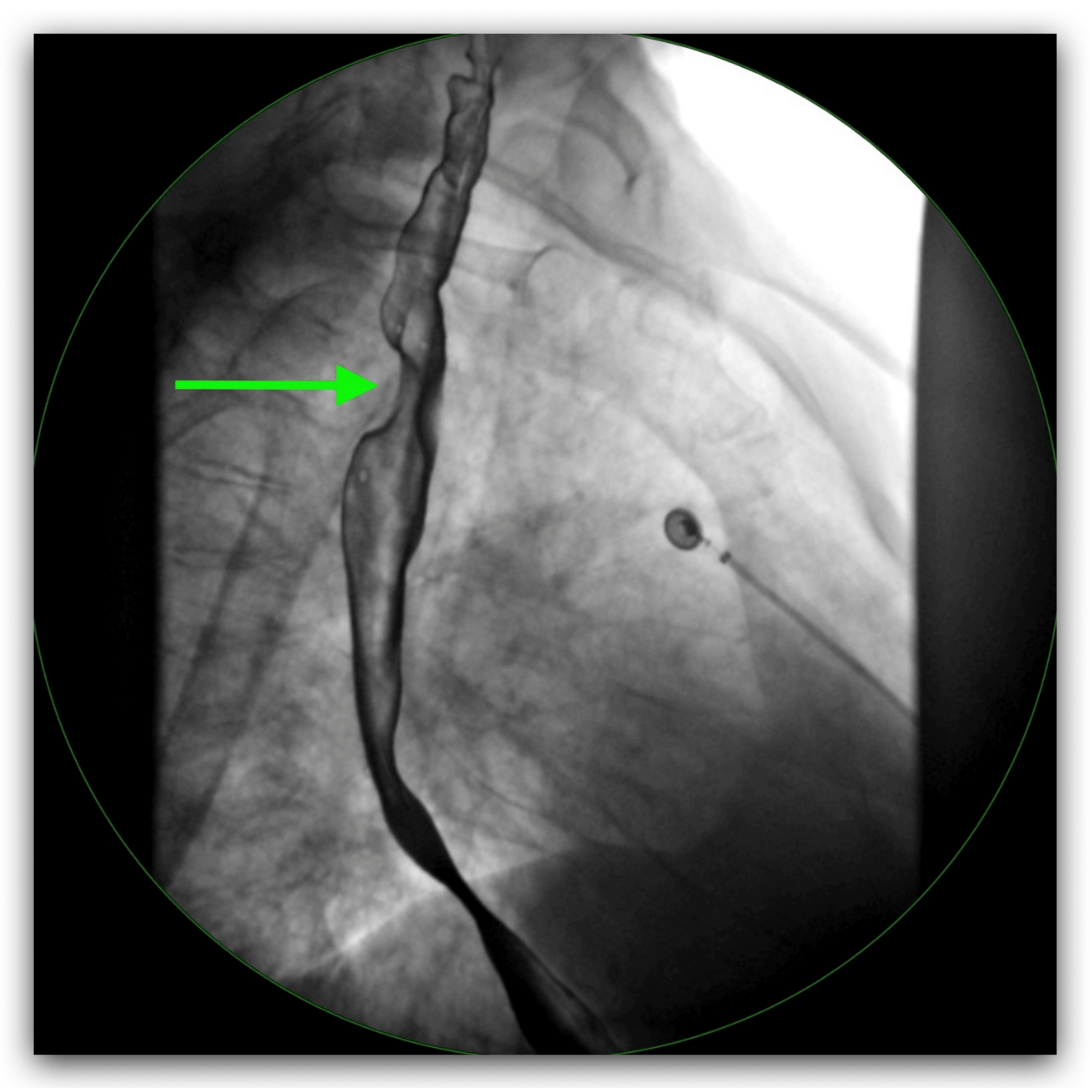
Upper gastrointestinal series X-ray with esophagram showed a small hiatal hernia, gastroesophageal reflux, and the green arrow indicates extrinsic compression of the lumen of the esophagus and the level of the subclavian artery

A chest X-ray was performed on admission and it showed diffuse interstitial prominence consistent with COPD (Figure 1). The patient was admitted to the hospital for further management with a diagnosis of COPD exacerbation. She was treated with oxygen via nasal cannula at 3 L per minute, albuterol-ipratropium was nebulized every six hours, and methylprednisolone 40 mg IV every eight hours. She persistently complained of dysphagia for solids. An electrocardiogram (EKG) showed sinus tachycardia with premature supraventricular complexes and left axis deviation (Figure 2). Because the patient had shortness of breath and there was concern for pulmonary embolism (PE), a D-dimer was ordered. Due to the elevated D-dimer, a CT angiogram (CTA) of the chest was performed, and it showed no evidence of PE but noted parenchymal abnormalities consistent with COPD (Figure 3). It also showed an aberrant origin of the right subclavian artery with an anomalous trajectory behind the trachea and the esophagus causing extrinsic compression of the esophagus. An esophagram was performed and it confirmed the extrinsic esophageal compression noticed in CTA of the chest (Figure 4). The patient was discharged from the hospital with a markedly improved respiratory status on albuterol-ipratropium nebulized every six hours and oral prednisolone in a tapering dose. She was advised to quit smoking and to follow up with her primary care provider. She was also prescribed pantoprazole 40 mg per mouth daily and instructed to avoid solid foods until evaluated by gastroenterology at a follow-up appointment.

## Discussion

The usual configuration of the large arteries arising from the arch of the aorta includes three main branches: the brachiocephalic trunk, which then divides into the right common carotid artery and the right subclavian artery; the left common carotid artery; and the left subclavian artery [2]. However, in the presence of an ARSA, the brachiocephalic trunk is absent. Instead, there are four large arteries arising from the arch of the aorta: the right common carotid artery, the left common carotid artery, the left subclavian artery, and the right subclavian artery [2]. The ARSA or right subclavian retroesophageal artery is a rare anatomical variation that occurs in approximately 0.5% to 2.5% of cases and is also known as "arteria lusoria" [3]. The prevalence of this anatomical variant varies across different geographic regions and patient populations. Studies have reported its occurrence in 0.5% of cases in North America, 0.1-0.2% in Asia, 0.16% in Greece, and 0.8% in Australia and Oceania [2]. Notably, certain patient demographics exhibit a higher prevalence of arteria lusoria. Myers et al. found a higher occurrence among women (55.3%) compared to men (44.7%) [3]. Furthermore, individuals with specific genetic disorders such as Down’s syndrome, Patau syndrome, and Edwards syndrome are more predisposed to this anomaly [4]. The right subclavian artery arises most distally from the aorta out of the four branches and crosses behind the esophagus and passes midline. There are three different possibilities of the anomaly: 80% run posterior to the esophagus, 15% run between esophagus and trachea, and 5% anterior to the trachea [5]. 

Arteria lusoria is typically asymptomatic because the aberrant artery does not form a complete vascular ring around the esophagus and trachea. Being typically asymptomatic, it is most often discovered incidentally [3]. Symptoms occur in a minority of cases and may include dysphagia (71.2%), dyspnea (18.7%), retrosternal pain (17%), cough (7.6%), and weight loss (5.9%) [2]. Compression of the esophagus by the right subclavian artery can lead to dysphagia, a condition also known as dysphagia lusoria [2]. Because the trachea is compressible in infants, they are more likely to present with respiratory symptoms such as wheezing, recurrent pneumonia, and cyanosis [2]. In adults, the trachea is more rigid, causing respiratory symptoms to be rare and dysphagia more common. Several mechanisms have been proposed to explain the symptoms in the adult population: 1) increased rigidity of the trachea; 2) aneurysm development, particularly when a Kommerell diverticulum (a congenital anomaly of the aortic arch) is present; 3) elongation of the aorta; and 4) increased stiffness of the artery wall due to atherosclerotic disease [2]. 

Arteria lusoria is typically diagnosed through non-invasive imaging modalities such as magnetic resonance angiography, CT of the chest, or CTA often in conjunction with an esophagram [5]. A chest X-ray can show widening of the superior mediastinum shadow [5]. CTA can be used to make a definitive diagnosis by identifying the aberrant origin of the right subclavian artery. In addition, an esophagram can be used for initial evaluation and it will show indentation of the posterior esophagus wall caused by the aberrant artery [5]. An esophagogastroduodenoscopy (EGD) to rule out concomitant causes of dysphagia may be considered. Medical management with dietary modification avoiding solid food and antacids as needed (PRN) are usually the first line choice. For those with severe symptoms that are refractory to medical management, surgery may be an option. Surgical treatment of arteria lusoria that has the lowest associated morbidity involves open ligation and transposition to the right carotid artery via a right supraclavicular approach [6]. 

## Conclusions

ARSA, also known as arteria lusoria, occurs when the right subclavian artery originates from the aortic arch. In rare instances, an ARSA can compress adjacent structures such as the esophagus resulting in dysphagia (dysphagia lusoria). In patients presenting with unexplained dysphagia, particularly to solids, clinicians should consider ARSA as part of the differential diagnosis. Imaging studies can help confirm the diagnosis, thus guiding clinical decisions and appropriate management. While many cases can be managed with dietary modifications, surgical intervention can be considered for patients with severe or refractory symptoms. Early recognition and appropriate treatment of dysphagia lusoria is crucial and can improve patient nutrition and overall quality of life.
